# The impact of work schedules, workplace bullying and some demographic characteristics on nurses’ sleep quality in Iran

**DOI:** 10.5935/1984-0063.20210029

**Published:** 2022

**Authors:** Masoomeh Najafzadeh, Kourosh Amini, Khosro Sadeghniiat-Haghighi, Koorosh Kamali

**Affiliations:** 1 Tehran University of Medical Sciences, Nursing and Midwifery Care Research Center, School of Nursing and Midwifery - Tehran - Tehran - Iran.; 2 Zanjan University of Medical Sciences, Social Determinants of Health Research Center - Zanjan - Zanjan - Iran.; 3 Tehran University of Medical Sciences, Occupational Sleep Research Center, Baharloo Hospital - Tehran - Tehran - Iran.; 4 Zanjan University of Medical Sciences, Social Determinants of Health Research Center - Zanjan - Zanjan - Iran.

**Keywords:** Shift Work Schedule, Bullying, Sleep Hygiene, Nurses

## Abstract

**Introduction:**

Sleep quality in nurses along with workplace bullying, are concerned with nursing care quality. There exist a few investigations on workplace bullying and its relationship with sleep quality. This study aims at determining the impact of work schedules, workplace bullying, and some demographic characteristics on nurses’ sleep quality.

**Material and Methods:**

This study was conducted on 333 nurses who worked in a hospital in Tehran, Iran. Sampling conducted from February 12 to April 23, 2020. Instruments of this study were Pittsburg sleep quality index and Quine’s workplace bullying scale.

**Results:**

63% of nurses had low sleep quality. Male nurses had lower sleep quality in comparison with females; 90% of nurses had encountered workplace bullying. Bullying and work schedules did not have a statistically significant effect on nurses’ sleep quality.

**Conclusion:**

According to this study, work schedules and workplace bullying had no significant effect on nurses’ sleep quality; but regarding that low sleep quality and encountering bullying is a cause of mental and physical problems for nurses and reduces the quality of care, it is recommended that nurses’ sleep quality and facing the bullying behavior should be taken into account by hospital authorities.

## INTRODUCTION

Nowadays, by changing the lifestyle, insomnia and sleep disorders are prevalent throughout the world; 5-50% of a studied population suffer somehow from sleep disorders^1^. Based on a recent study in Belgium, about 40% of nurses have sleep disorders^2^.

Previous studies have shown that sleep disorder in nurses, especially the low quality of sleep, reduces the safety of nurse and patient, in addition to other problems in their physical and mental health, and overshadows the nursing cares^3-5^. Some of the physical and mental complications of insomnia are fatigue and boredom, depression, burnout, reducing motivation, mental-physical disorders, reducing attention, and being wide-awake, change in mood and being ill-tempered, gastrointestinal, and musculoskeletal disorders^6-8^. In addition, studies have shown that bullying is one of the common psychological problems in the workplace. Bullying is defined as causing horror, insult, social deprivation, and negative effects on the performance of an individual or a group, provided that this behavior occurs repeatedly and regularly during a long time (for example, six months)^9^. Bullying victims are terrified, annoyed, ignored, humiliated, deprived of resources and facilities, and isolated. They are prevented from defending their rights. This group of people has low job satisfaction, weak job performance, and low motivation and job efficiency. Furthermore, bullying can have a negative effect on the intra- and extra-organizational social communications. For example, nurse bullying and other disruptive behaviors impede communication, which ultimately have a negative impact on essential information being shared between healthcare workers^9,10^.

The evidences show that bullying in the workplace, not only has negative effects on the victims, but also increases the costs of providing human resources in organizations, which are caused by absence, desertion, and turnover^7^. It seems that the importance of this dilemma is two-fold in the nurses, because such improper behaviors will influence potentially the care level and safety of patients^11^.

Literature review indicates that various descriptive studies have been conducted about the sleep disorders of nurses up to now^2-5^. Regarding some weaknesses in the previous researches, such as limitations in finding generalizability, it is recommended that these studies should be repeated in other societies^12,13^. Moreover, in studies, some contradicted results are reported about the effective factors on the sleep of nurses^14^. On the other hand, there are few studies about the influence of bullying on the nurses’ sleep and it is not possible to come to a definite conclusion based on them^14^. In addition, following the changes occurred by performing the nurses’ shift work productivity plan and obligatory shift rotation in Baharloo hospital, it is suggested that it requires further research.

With regard to above mentioned issues and the crucial point that patients’ safety is one of the essential concepts in healthcare systems, and its correlation with nurses’ sleep quality and encountering bullying in the workplace; this study was conducted in order to determine the impact of work schedules, workplace bullying, and some demographic characteristics (gender, age, education, marital status, having second job, and number of children) on nurses’ sleep quality.

## MATERIAL AND METHODS

### Study design

This research was a case-control study that investigates the effect of work schedules and encountering workplace bullying on the sleep quality of nurses in a hospital affiliated to Tehran Medical Sciences University. Sampling conducted from February 12 to April 23, 2020.

### Participants

**Figure 1:**
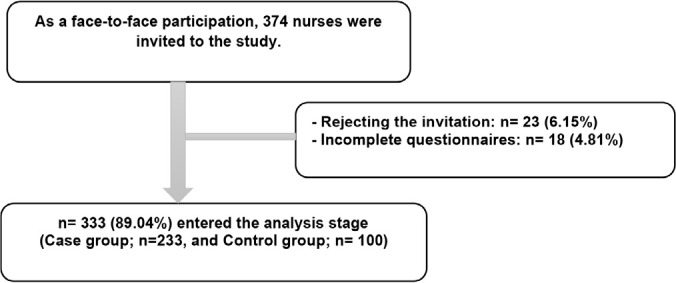
Flowchart of participants’ recruitment.

The study population were all the nurses who worked in the hospital. Participants had the inclusion criteria for the study, which comprises: (a) tendency to participate in the research and signing informed consent form; (b) lack of physical and mental diseases in participants; (c) not being in critical conditions such as losing a beloved one; (d) at least 6-month work experience in nursing; (e) non-pregnancy; and (f) non-addiction. The number of nurses working in the hospital at the time of the study was 374. From among them, 41 nurses (10.96%) were reluctant to participate in the study. Nurses with low sleep quality (Pittsburg sleep quality index score >5) were determined and placed in the case group. Nurses with fine sleep quality (Pittsburg sleep quality index score ≤5) were also identified and put in the control group. The summary of enrolling the nurses in the study is shown in Figure 1.

### Instruments

Demographic information questionnaire, Pittsburg sleep quality index (PSQI) and Quine’s bullying in workplace questionnaire were used. PSQI is a standardized self-report questionnaire that indicates the quality of sleep during the recent month. This questionnaire has been used in various national and international researches and is one of the best tools designed for measuring sleep quality. PSQI was made in 1989, by Buysse et al.^15^, in Pittsburg psychological institute. This index has 9 items but five of its questions have 10 sub-items; therefore, the total items of the index are 19 that are scored in a 4-point Likert scale from 0 to 4.

PSQI have 7 subscales including: 1 - subjective sleep quality; 2 - sleep latency; 3 - sleep duration; 4-habitual sleep efficiency; 5 - sleep disturbances; 6 - use of sleeping medications; 7 - daytime dysfunction. The results of this questionnaire are scored from 0 to 21. The 0-5 score indicates good sleep quality, 6-10 is relatively low sleep quality and 11-21 is low sleep quality^15^.

The validity and reliability of PSQI have been measured^16^. Although the above classification was used to determine those who have sleep quality disorders, but in the final analysis of data, the mean obtained score of participants was utilized, which showed the mean global scores of the participants in the case and control groups.Quine’s bullying in workplace questionnaire was used to measure workplace bullying which has 5 parts; the first part of the questionnaire is about threatening the job position and includes 4 questions; the second part is about threatening the individual position and includes 7 questions; the third part is related to the isolating and has 3 questions; the fourth part is about high workload with 2 questions and the fifth part is related to instability with 4 questions. All 20 questions have yes/no answers and if the answer of the participant to 1 question of 20 questions is yes, he/she is under bullying^12^. It is worthy to mention that Quine’s bullying in workplace questionnaire in Iran is not standardized. In our study, translation/back-translation along with expert panel were used to determine the validity of the questionnaire. Cronbach alpha (α=0.796) was measured to confirm the internal consistency. Reliability was also determined by test-retest. Furthermore, the reliability and validity of this scale have been demonstrated in previous studies^17,18^. It should be noted that the authors received this questionnaire through e-mail correspondence with the toolmaker and there is no limitation for using it.

### Data collection

Necessary permissions were taken from the authorities of Tehran Medical Sciences University and Baharloo hospital to conduct the study. Nursing managers, educational and research supervisors were coordinated to participate in this research. In the next step, the researcher had meetings with head nurses and nurses of all hospital wards in different work shifts and explained the objective of the research and the procedure, and answered their questions to remove all ambiguities. After obtaining written informed consent forms, demographic, and job information questionnaires, as well as Pittsburg sleep quality index (PSQI) were distributed among nursing personnel. Then, Quine’s bullying in workplace questionnaire was distributed among nurses of both groups, and the last step was statistical analysis. It is necessary to mention that the work schedule was considered as a job characteristic in the demographic questionnaire.

### Ethical considerations

1.) Obtaining ethics committees permission of Tehran and Zanjan Medical Sciences Universities (Ethical code: ZUMS.REC.1395.110); 2.) Obtaining permission of hospital authorities to start the research; 3.) Informing the participants about the purpose of the study; 4.) Obtaining informed written consent form for participation in the research; 5.) Preserving the anonymity of subjects in the research process; 6.) Giving the research subjects authority to non-participation in the research; 7.) Emphasizing on the subjects’ access to the findings after its completion.

### Data analysis

**Figure 2:**
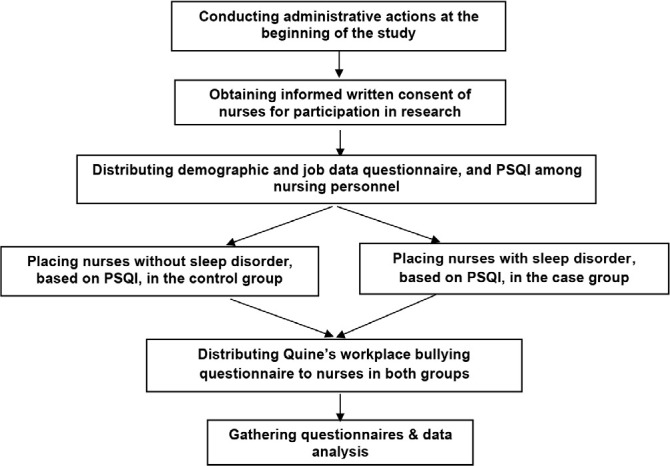
Research process flowchart.

To perform data analysis, SPSS software version 16 was employed. Descriptive statistics was used to measure the characteristics of research subjects. Chi-square and independent t-test were applied to study the homogeneity of both groups, based on important predictor variables (age, gender, and marital status). Chi-square test was used to compare both groups in terms of the frequency of encountering workplace bullying and having fixed or rotational shifts. In addition, multivariable logistic regression analysis was brought into operation to assess the effect of the predictor variables on sleep quality, while adjusting potential confounder effects. The *p*≤0.05 was considered significant. Process of the study is shown in Figure 2.

## RESULTS

From among 333 nurses who participated in the study, 69.9% had problems in the quality and quantity of sleep (case group); 90% of all the nurses who took part in this survey had encountered workplace bullying. Regarding demographics, most participants in both case (80%) and control (79.8%) groups were female. About 77% of nurses in both groups had bachelor degree. 63.2% in the case group and 30% in the control group were single; 50% in the control group were recruited full-time but this ratio was 35.6% in the case group. Most participants in the case group (78.1%) and control group (89%) were physically healthy. Most nurses in case group (86.3%) and control group (86%) worked in the hospital only with one job (nursing). About 24% of nurses in both groups had only one job. Only 13.7% of nurses in the case group and 14% in control group were under treatment with medicine. 15.9% of participants in the case group and 15% in the control group had close old or patient relatives at home who need special care. Mean and standard deviation of nurses’ age in case and control groups were 30.67±6.07 and 32.36±5.50, respectively. BMI mean and standard deviation of nurses in the case and control groups were 23.99±5.04 and 24.32±3.82, respectively. Mean and standard deviation of job experience of nurses were 6.55±5.04 in the case group, and 8.56±5.26 in control group. Mean and standard deviation of the number of children were 0.60±0.8 and 0.79±0.83 in case and control groups, respectively. Shapiro Wilk test was used to check if the quantitative results were normally distributed. It should be mentioned that case and control groups had a significant statistical difference in terms of marital status, recruitment type, age, and years of service (*p*<0.05). But there was no significant statistical difference between the two groups regarding gender, education, physical health, having the second job, taking medicine, BMI, etc., (*p*>0.05). The demographic characteristics of subjects in both case and control groups were described in Table 1.

**Table 1:** Descriptive characteristics of population in cases and controls.

Socio-demographic characteristic	Cases (n=233)	Controls (n=100)	p-value
Gender			
Female	186(79.8%)	84(84%)	0.373
Male	47(20.2%)	16(16%)	
Degree of education			
Diploma	39(16.7%)	15(15%)	0.158
Associate	9(3.8%)	2(2%)	
BSc	180(77.4%)	77(77%)	
MSc	5(2.1%)	6(6%)	
Marital status			
Married	87(37.3%)	65(65%)	0.008
Single	145(62.2%)	30(30%)	
Divorced	1(0.5%)	5(5%)	
Physical health			
Yes	203(87.1%)	89(89%)	0.633
No	30(12.9%)	11(11%)	
Having a second job			
Yes	40(17.2%)	14(14%)	0.493
No	193(82.8%)	86(86%)	
Taking any medication			
Yes	32(13.8%)	13(13%)	0.949
No	201(86.2%)	87(87%)	
Presence of a sick or elderly person at home			
Yes	37(15.9%)	15(15%)	0.827
No	196(84.1%)	85(85%)	
Age (Years), Mean ± SD	30.67 ± 6.07	32.36 ± 5.50	0.018
Body mass index, Mean ± SD	23.99 ± 3.84	24.32 ± 3.82	0.482
Work experience in nursing (years), Mean ± SD	6.55 ± 5.04	8.56 ± 5.26	0.001
Number of children, Mean ± SD	0.60 ± 0.82	0.79 ± 0.83	0.059

While a logistic regression model has a binary outcome and one predictor, a multivariate logistic regression (backward method) analysis finds the equation that best predicts the success value of the binary response for the values of several predictor variables. Adjusted odds ratio is an odds ratio that controls for other predictor variables in the model and gives an idea of the dynamics between the predictors. According to Table 2, the adjusted odds ratio was calculated for sleep quality factors based on multivariable logistic regression. The results showed that the highest odd ratio belongs to males. No significant relationship was reported between other variables of the study and sleep quality. Findings of the study demonstrated that the prevalence of encountering workplace bullying in the case group was not statistically different with the control group [90% vs. 86.6%, OR=1.85 (95% CI: 0.66-5), *p*=0.240]. In addition, there was no significant statistical difference in rotating shifts between case and control groups [90.5% vs. 81.0%, OR=1.35 (95% CI: 0.57-1.2), *p*=0.495].

**Table 2:** Adjusted odds based on multivariable logistic regression for risk factors of sleep quality.

Parameters in regression models	OR (95% CI - OR)	p-value
Encountering workplace bullying	1.85(0.66-5)	0.240
Male gender compared to female	2.85(1.4-5.7)	0.003
Age	1.05(0.99-1.1)	0.067
Working shifts rotation in comparison with fixed shifts	1.35(0.57-1.2)	0.495

## DISCUSSION

This study was conducted to determine the impact of work schedules, workplace bullying, and some demographic characteristics on nurses’ sleep quality. The data analysis showed that there was no significant statistical difference comparing fixed and rotating work schedules and encountering bullying behaviors in both groups (case and control); and both studied variables had no effect on the sleep of nurses. Few papers are published^19,20^ about the effect of encountering workplace bullying on nurses’ sleep quality. In a study by Ovayolu et al. (2014)^19^, in Turkey, the results showed that 66.2% of nurses who encountered bullying behaviors in the workplace had health problems or sleep disorder. Niedhammer et al. (2009)^20^ argued that encountering bullying behaviors is related to sleep disorders in France. Not only encountering workplace bullying in the past can impair sleep quality, but also the frequency of exposure to bullying at work is also associated with worse sleep quality. Even observing those who are exposed to workplace bullying can lead to sleep disorder^20^. Regarding the different results of our study compared to previous studies, we can refer to the role and importance of sociocultural or contextual factors. According to the studies, factors such as management styles^21^, number of personnel or nurse to bed ratio^22^, physical condition of workplace^23^, mental atmosphere of workplace^24^, and psychological and personality differences^25^ can also have an influence on sleep quality and quantity. Another important point, that can be effective on getting our different results compared to previous studies, is the high prevalence of sleep disorder among nurses in our study that disturbed the balance of the number of samples in case and control groups.

Other studies confirm our findings of the lack of relationship between the quality of sleep with rotational work shifts^14,26^. Chien et al. (2013)^14^ demonstrated that nurses with an educational level of high school or under, have two times higher incidence of poor sleep quality when compared with nurses with an educational level of college or higher degree. Nurses with higher educational level have a less work-related stress and less emotional exhaustion. It is not identified if nurses with higher education have leadership or coordination responsibilities^14^. However, there are opposite findings in some researches^23,27,28^, which shows type of shifts and turns are effective on the sleep quality. It seems that in the previous studies (especially those that were conducted in Iran), the difference in the results can be attributed to the sample size. In addition, pregnant nurses or nurses with diagnosed mental and physical diseases were excluded from the survey. These important factors in sleep quality were not considered in previous studies. However, it seems that factors which are more important than rotational work shifts and encounter workplace bullying play a seminal role in sleep quality, and it needs further research.

Another interesting finding indicated that 63.3% of nurses had low sleep quality. The previous studies showed that sleep disorder is one of the most common complaints of those who work in shifts, especially nurses^29^. Some other researches also showed that nurses’ rotational and long-term shifts and their family responsibilities are among the major causes of this high prevalence of sleep disorders^30^. These factors, in addition to causing physical and psychological problems, lead to low job satisfaction, weak job performance, low productivity, and reduce the quality of service providing and safety of nurse and patient^3-5^.

Regarding the frequency of encountering workplace bullying in this study, most nurses had experienced this behavior at work (90%). The frequency of encountering workplace bullying in the studied nurses is significantly higher than other research societies. In previous studies, encountering workplace bullying is a common problem and a cause of increasing the costs of providing human resources due to the absence and turnover of nurses^7^. This finding is consistent with Longo (2013)^11^ study, who concluded almost all nurses encounter bullying behavior during their working life. In a study performed by Allen et al. (2014)^30^, 61% of nurses had experienced workplace bullying. Yildirim (2009)^31^ in Turkey discussed that 37% of nurses had never encountered bullying behavior and only 21% were exposed to this behavior. It seems that culture and cultural context play an important role in the frequency of encountering this phenomenon that should be studied in other researches.

Another interesting finding of this study was that men have lower sleep quality than women. Reviewing the studies about the effect or relationship of gender with quality of sleep or sleep disorders shows contradictory results. Some studies show that there is no relationship between gender and sleep^32,33^. Other surveys conclude that women suffer more than men from sleep disorders^34,35^. However, high frequency of sleep disorder among men in this study can be related to the second job. Compared with females, male nurses have to earn extra money for life expenses. It should be mentioned that the traditional role of man as the bread earner of the family is socially accepted in Iran.

In conclusion, the results of this study indicated that encountering workplace bullying is a common experience in most of the studied nurses and the majority of them do not have acceptable sleep quality. However, the results demonstrated that rotational shifts and encountering bullying behavior do not have a significant role in the nurses’ quality of sleep.

### Strengths and limitations

This investigation comes with its own set of strengths and weaknesses. The first strong aspect is its case-control design. The second one concerns with the large sample size. The third feature is the use of PSQI to measure the dependent variable that its reliability and validity have been confirmed in various studies. PSQI is assumed to be a powerful tool to identify the sleep quality of various working groups, especially nurses. The down aspect of current study is its restricted population in one hospital that reduces its generalizability. Another weakness of this research, that was out of researchers’ control and influenced the findings, is the not equal number of samples in the case and control groups. As it was cited earlier, the majority of the sample population had encountered bullying and most of them had rotating shift schedules. It is recommended that other future studies with higher numbers of participants should be conducted to control this limitation.

Regarding the importance of the nursing profession and their responsibility for physical and mental health of patients, and with respect to the effect of low sleep quality and encountering workplace bullying on health and safety of patients and nurses and the quality of care, it seems that further research is required. Since the results of this study indicated that most nurses have low sleep quality and encountered workplace bullying, these problems should be considered by nursing and hospital managers in clinics, wards and hospitals, and some measures should be taken to reduce them. It is suggested that nurses should be evaluated in terms of sleep disorder and encountering workplace bullying, as they are evaluated for illnesses and other annual work related problems through occupational medicine.
